# Photocatalytic Synthesis of Materials for Regenerative Medicine Using Complex Oxides with β-pyrochlore Structure

**DOI:** 10.3390/life13020352

**Published:** 2023-01-28

**Authors:** Ludmila Semenycheva, Victoria Chasova, Diana Fukina, Andrey Koryagin, Artem Belousov, Natalia Valetova, Evgeny Suleimanov

**Affiliations:** The Research Institute for Chemistry, Lobachevsky State University of Nizhny Novgorod, pr. Gagarina 23, Nizhny Novgorod 603950, Russia

**Keywords:** cod collagen, methyl methacrylate, complex oxides, RbTe_1.5_W_0.5_O_6_, CsTeMoO_6_, RbNbTeO_6_, β-pyrochlores, photocatalysis, graft copolymer, enzymatic hydrolysis, scaffold

## Abstract

Graft copolymerization of methyl methacrylate onto cod collagen was carried out under visible light irradiation (λ = 400–700 nm) at 20–25 °C using the RbTe_1.5_W_0.5_O_6_, CsTeMoO_6_, and RbNbTeO_6_ complex oxides with β-pyrochlore structure as photocatalysts. The as-prepared materials were characterized by X-ray diffraction, scanning electron microscopy, and UV-Vis diffuse reflectance spectroscopy. It was also found that RbNbTeO_6_ with β-pyrochlore structure was not able to photocatalyze the reaction. Enzymatic hydrolysis of the obtained graft copolymers proceeds with the formation of peptides with a molecular weight (MW) of about 20 and 10 kDa. In contrast to collagen, which decomposes predominantly to peptides with MW of about 10 kDa, the ratio of fractions with MW of about 10 kDa and 20 kDa differs much less, their changes are symbatic, and the content of polymers with MW of more than 20 kDa is about 70% after 1 h in the case of graft copolymers. The data obtained indicate that synthetic fragments grafted to the collagen macromolecule do not prevent the hydrolysis of the peptide bonds but change the rate of polymer degradation. This is important for creating network matrix scaffolds based on graft copolymers by cross-linking peptides, which are products of enzymatic hydrolysis.

## 1. Introduction

The creation of new generation materials with required performances and high reliability is inextricably linked with the use of new approaches. Much attention in recent years has been paid to investigations related to the principles of green chemistry. Photocatalytic complexes based on metal oxides are an environmentally friendly alternative to chemical sources of active particles in the conversion of various chemicals [1,2]. Their application makes it possible to avoid the use of auxiliary and intermediate substances in the synthesis, reduces the number of process steps, etc. Different photocatalytic reactions, such as water splitting [3,4,5,6,7], the degradation of organic substances in aqueous solutions [8,9,10,11,12,13,14,15,16,17,18,19,20], and polymerization [21,22,23,24], have been intensively studied. The use of photocatalysis for obtaining biomedical materials based on natural polymers is an especially attractive area of research. In this case, an environmental aspect, i.e., the use of renewable feedstocks, is considered.

Theoretical and experimental studies that make it possible to develop fundamentally new ways to obtain the creation and application of environmentally friendly technologies with the required properties are an urgent task. An actively developed strategy in this direction is the development of compositions based on large-scale synthetic polymers and natural origin polymers. The main advantage of these materials is the unique combination of the component properties assembled in a certain structure. One of the most important consumers of such materials is regenerative medicine associated with the stimulation of cellular/tissue regeneration [23,24,25,26,27,28,29,30,31,32,33,34]. Regenerative medicine is a new stage in the evolutionary development of medical technologies, which arose at the intersection of medicine, biology, physics, chemistry, etc. This makes it possible to classify this branch of medicine, which uses modern achievements in each scientific field, as an interdisciplinary type of scientific and practical activity.

It is well-known that several reactive oxygen species (ROS), including highly-active hydroxyl radicals (^•^OH), are formed in aqueous solutions in the presence of metal oxides under ultraviolet or visible light irradiation [35]. Hydroxyl radicals are capable of initiating polymerization with the participation of monomeric and polymeric molecules, including the grafting of a synthetic monomer onto a natural origin polymer. This process has been studied primarily for titanium oxide (TiO_2_) as a photocatalyst [19,22].

In our previous studies, graft copolymers of acrylic monomers onto cod collagen (CC) and pectin have been prepared using the RbTe_1.5_W_0.5_O_6_ photocatalyst under visible light irradiation (λ = 400–700 nm) at 20–25 °C [25,26]. The visualized morphology of lyophilically dried samples of graft copolymers of alkyl (meth)acrylates with CC indicates their well-defined three-dimensional (3D) network structure. This proves their obvious promise for the use as scaffolds in regenerative medicine. It is well-known that proteolytic enzymes are often included in the initial reagents for the preparation of scaffolds containing proteins [36,37,38,39,40,41,42,43,44,45]. This leads to the hydrolysis of the peptide bond of proteins, and the resulting peptides with lower MW compared to the original proteins form new structural structures, namely scaffolds. Obviously, the enzymatic hydrolysis of proteins plays an important role in the formation of scaffolds. Previously, we have shown that during the enzymatic hydrolysis of CC, the destruction of a high molecular weight polymer to low molecular weight fragments occurs very quickly, almost within the first minute at room temperature [34,46,47,48,49]. During the enzymatic hydrolysis of CC together with fibrinogen responsible for the formation of the network structure, a scaffold is formed, which has a whole range of necessary biomimetic properties [36,37]. Graft copolymers of alkyl (meth)acrylates with CC are supposed to be used in scaffolds to create a framework like that in scaffolds with fibrinogen. In this regard, the change in their MW parameters in the presence of enzymes will play a primary role.

The main goal of this work is to reveal the reasons of the changes in MW parameters during the enzymatic hydrolysis of graft copolymers of methyl methacrylate (GCMC) with cod collagen, as well as to compare them with those for CC to identify the conditions to obtain scaffolds based on them. The fragments of GCMC formed during enzymatic hydrolysis with lower MW compared to the initial macromolecules should become the structural elements of scaffolds. We have previously shown that during the enzymatic hydrolysis of CC and fibrinogen under comparable conditions, the MW values of both the initial fibrinogen and CC samples and the fractions of CC and fibrinogen hydrolysates have similar values [36]. This has allowed for the suggestion of a scheme for the formation of the scaffold. During the proteolysis of fibrinogen by the enzyme thrombin, a fibrin monomer is formed, which consequently forms a dimer, tetramer, octamer, etc., when interacting with similar fibrin monomers. As a result, a large molecular complex, a so-called “soluble fibrin” or “soluble fibrin monomer complexes”, is formed [50]. According to the literature [50], the MW of this complex is about 12,000–15,000 kDa. During the simultaneous hydrolysis of fibrinogen and CC at the stage of fibrin polymer formation, macromolecules of CC hydrolysate are incorporated into the framework, forming a spatial scaffold structure [36]. In this work, graft copolymers were obtained using not only the RbTe_1.5_W_0.5_O_6_ complex oxide with β-pyrochlore structure as a photocatalyst [26], but also its analogs, namely CsTeMoO_6_ and RbNbTeO_6_. The use of other complex oxides as photocatalysts was studied to reveal the influence of the nature of β-pyrochlore oxide on the composition and ratio of the protein and synthetic parts of graft copolymers.

## 2. Materials and Methods

### 2.1. Materials

Commercial reagents, namely acetic acid, sodium hydroxide, and proteolytic enzyme (pancreatin with protease activity of 2 U/mL), were used in this work. Methyl methacrylate (MMA) was used as a monomer. The monomer was preliminarily purified from the stabilizer by washing with a sodium hydroxide solution, then it was washed with cold water to a neutral pH, dried with calcium chloride, and distilled in vacuum (1.33 Pa) at 40 °C.

### 2.2. Preparation of Photocatalysts

The RbTe_1.5_W_0.5_O_6_, CsTeMoO_6_, and RbNbTeO_6_ photocatalysts were prepared via a solid-state reaction, as described in previous works [13,51]. Briefly, stoichiometric amounts of CsNO_3_ (99%), RbNO_3_ (99%), TeO_2_ (99%), MoO_3_ (99%), WO_3_ (99%), or Nb_2_O_5_ (99%) were mixed in an agate mortar and sintered at 700–750 °C for 24 h in a platinum crucible at a heating rate of 5 °C/min. The prepared powders were then grinded by ball milling for 18 h with a speed of 300 min^−1^ using isopropyl alcohol as a process control agent.

### 2.3. Characterization of Photocatalysts

XRD patterns were recorded on a Shimadzu XRD-6100 diffractometer (CuKα radiation λ = 1,5418 Å) with a Ni filter (2θ = 10–80°, 1°/min). The morphology of the samples was studied by SEM on a JSM-IT300LV microscope (JEOL). The elemental composition was proven by energy dispersive X-ray spectroscopy (EDX) equipped with an X-maxN 20 detector (Oxford Instruments). The particle size distribution by volume of the samples was investigated using a laser diffraction particle size analyzer SALD-2300 (Shimadzu, Japan). UV-Vis diffuse reflectance spectra (DRS) were obtained on a Cary 5000 spectrophotometer (Varian, Canada). The optical band gaps (*E*_g_) of the as-prepared materials were determined using Kubelka-Munk theory.

The details of physicochemical properties of the prepared materials are presented in our previous works [15,51].

### 2.4. Isolation of Collagen from Integumentary Tissues of Cod

Collagen was isolated by extraction with acetic acid for a day at room temperature according to the method described previously [52]. The resulting acetic acid dispersion was dried to constant weight under vacuum (1.33 Pa) at 50 °C.

### 2.5. Photocatalytic Synthesis of Graft Copolymers MMA–Collagen using Complex Oxides with β-pyrochlore Structure

For the photocatalytic experiments, an emulsion was prepared by mixing a 5% solution of CC with MMA in a 2:1 volume ratio. In this case, collagen acts not only as a reagent, but also as an emulsifier. A stable emulsion is formed at a ratio of 2:1 and intensive mixing. The photocatalysts were added in a mass ratio of emulsion/photocatalyst of 100:1, then argon was bubbled through the reaction mixture for 15 min. The resulting emulsion was stirred at a speed rate of 600 rpm and irradiated using a 30 W LED lamp in an argon flow for 5 h. After polymerization, the reaction mixture was centrifuged to remove the photocatalyst. For extracting the organic phase, 10 mL of toluene was added to the emulsion followed by stirring at a speed rate of 600 rpm for 3 h. The photocatalysts were washed several times with water and dried at 50 °C and 1.33 Pa to constant mass.

### 2.6. Enzymatic Hydrolysis of Graft Copolymers

Hydrolysis with the proteolytic enzyme, pancreatin, was carried out at room temperature and at a pH of about 7.0 (the ratio of graft copolymer/pancreatin of 10^3^:1). To stop the reaction, a 4% solution of acetic acid was added to the reaction mixture in a volume ratio of 1:1.

### 2.7. Analysis of Molecular Weight Characteristics

Molecular weight characteristics were determined by gel permeation chromatography (GPC). Organic solutions in tetrahydrofuran were analyzed using a liquid chromatograph “Shimadzu Prominence LC-20VP” with “Tosoh Bioscience” columns (eluent flow rate of 0.7 mL/min). Narrow disperse polystyrene standards were used for calibration; a differential refractometer was used as a detector. Water solutions were analyzed using a high-performance liquid chromatograph manufactured by Shimadzu CTO 20A/20A C (Japan) with the LC-Solutions-GPC software module. Separation was performed using a Tosoh Bioscience TSK gel g3000swxl column with a pore diameter of 5 μm and a low-temperature light-scattering detector ELSD-LT II. 0.5 M acetic acid solution was used as an eluent with a flow rate of 0.8 mL/min. Narrow disperse dextran standards with a molecular weight range of 1–410 kDa (Fluca) were used for calibration.

### 2.8. Scanning Electron Microscopy

The study of the surface of proteins, scaffolds, and polymer samples was performed using a scanning electron microscope (SEM) JSM-IT300 (Jeolltd, Japan) with an electron probe diameter of 5 nm (operating voltage 20 kV), using detectors of low-energy secondary electrons and backscattered electrons in a low vacuum mode to avoid samples charging. An elemental composition investigation of the photocatalyst powder was performed on method energy dispersive X-ray microanalysis (EDXMA) with the detector X-Max^N^ 20 (Oxford Instruments).

### 2.9. Elemental Analysis of Polymers

Samples were analyzed by CHNS analysis on an elemental analyzer Vario EL cube for the simultaneous determination of CHNS(O).

### 2.10. Lyophilic Drying

The sample sponges were obtained by lyophilic drying. The distillation of the solvent was carried out under vacuum (1.33 Pa) under deep freezing using liquid nitrogen.

## 3. Results and Discussion

### 3.1. Change in the Characteristics of Graft Copolymers Methyl Methacrylate with Cod Collagen, Synthesized Using Complex Oxides with β-pyrochlore Structure as Photocatalysts under Visible Light Irradiation, during Enzymatic Hydrolysis

#### 3.1.1. Effect of Cod Collagen Drying on the Enzymatic Hydrolysis with Pancreatin

The modification of the polymer macromolecule structures in solutions using enzymes is a well-known tool for controlling the process of structure formation and obtaining porous hydrogel matrices for regenerative medicine [36,37,38,39,40,41,42,43,44,45]. In this study, for the transformation of the polymer via the enzymatic hydrolysis by the proteolytic enzyme, pancreatin, GCMC, which were synthesized using complex oxides with β-pyrochlore structure as photocatalysts under visible light irradiation, were chosen. It is known that collagen scaffolds have a low strength and high biodegradation rate, which prevents their wide application [53]. Due to the inclusion of synthetic polymer fragments in the fibrillar organization of collagen and the corresponding strengthening of the matrix structure, GCMC is considered as a promising material to produce scaffolds with a good strength and moderate biodegradation rate.

It has been previously found that native CC, isolated from the integumentary tissues of cod in an acetic acid solution (MW of about 300 kDa), after neutralization and the addition of catalytic amounts of proteolytic enzymes (mass ratio collagen/pancreatin of 103:1–102:1), noticeably decomposes to low molecular weight fragments with MW of about 10 kDa and 20 kDa within a few minutes under standard conditions. Hydrolysis continues until the complete disappearance of the high molecular weight fraction for several hours in the subsequent control period.

Firstly, it is necessary to clarify how the lyophilic drying of the CC solution will affect the results of enzymatic hydrolysis. Obviously, this collagen has a much longer shelf life than in an acetic acid solution, where it gradually undergoes partial hydrolysis. Therefore, this collagen was used to obtain GCMC.

**Figure 1:**
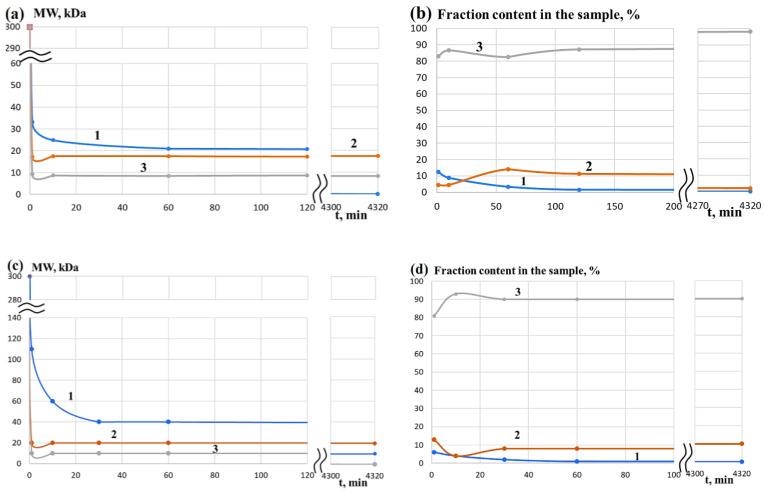
Changes in the molecular weight characteristics of different fractions during hydrolysis of a solution (**a**,**b**) [49] and dried collagen (**c**,**d**) with pancreatin: 1—fraction with MW above 20 kDa; 2—fraction with MW of about 20 kDa; 3—fraction with MW of about 10 kDa.

Changes in the molecular weight characteristics of the collagen (DCC) that was dried and redissolved in water were monitored for three days. It was observed that DCC is destroyed in the presence of pancreatin in almost the same way as CC after its neutralization with an acetic acid solution (Figure 1), and the fraction with a MW of about 300 kDa completely disappears within a few minutes.

Thus, it could be concluded that the data on CC and DCC hydrolysis are comparable.

#### 3.1.2. Effect of the Nature of the Complex Oxide with β-pyrochlore Structure Used as Photocatalysts of Graft Copolymerization on the Composition and Structure of Graft Copolymers of Methyl Methacrylate with Cod Collagen

**Figure 2:**
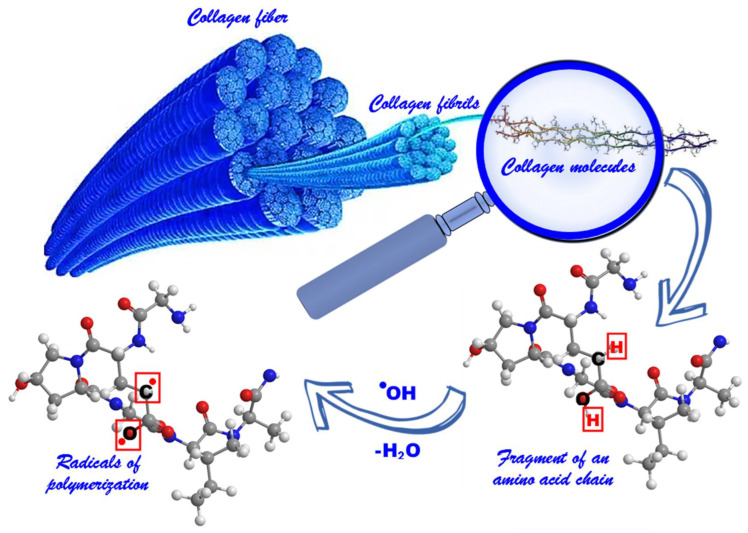
Scheme of the formation of polymerization radicals.

The synthesis of GCMC graft copolymer samples was carried out using the RbTe_1.5_W_0.5_O_6_, CsTeMoO_6_, and RbNbTeO_6_ complex oxides as photocatalysts under visible light irradiation (λ = 400–700 nm) at 20–25 °C. During light irradiation, electron-hole pairs are formed, which could lead to a series of transformations with the formation of hydroxyl radicals [15,54]. The interaction of hydroxyl radicals with hydrocarbon hydroxyl fragments of collagen (Figure 2) leads to the formation of radicals on the surface of the protein. Due to the presence of these radicals, polymethyl methacrylate (PMMA) is grafted onto collagen [47].

It is noteworthy that the formation of noticeable amounts of graft copolymer took place only in the case of RbTe_1.5_W_0.5_O_6_ and CsTeMoO_6_, which were denoted as GCMC–1 and GCMC–2, respectively. The formation of polymers in the presence of the RbTe_1.5_W_0.5_O_6_ and CsTeMoO_6_ photocatalysts is evidenced by the analysis of the polymer product isolated from the aqueous phase. The molecular weight of the samples increased markedly from 240 to 270 kDa and 280 kDa for GCMC–1 and GCMC–2, respectively. At the same time, the polydispersity coefficient did not change and amounted to 1.2. The collagen isolated after synthesis using the RbNbTeO_6_ complex oxide did not change its molecular weight parameters.

**Figure 3:**
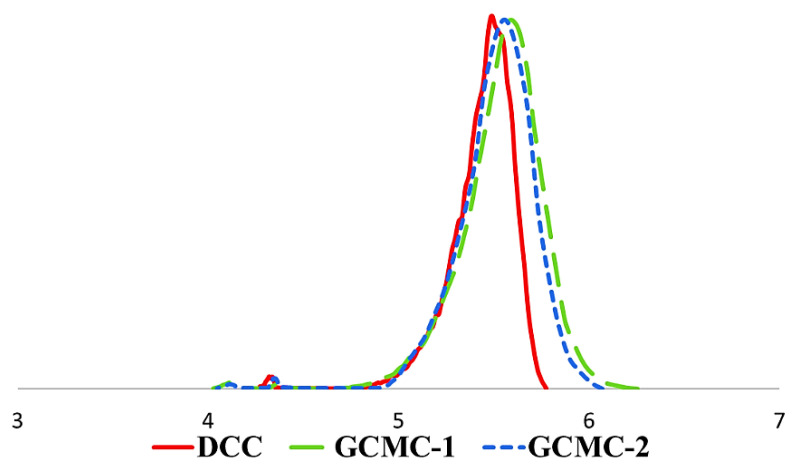
The MWD curves of initial dried collagen (DCC), PMMA-collagen graft copolymer after photocatalysis using RbTe_1.5_W_0.5_O_6_ (GCMC–1) and CsTeMoO_6_ (GCMC–2).

As can be seen from Figure 3, there is a shift in the molecular weight distribution (MWD) to the high molecular weight region, which is obvious evidence of MMA grafting onto collagen over the RbTe_1.5_W_0.5_O_6_ and CsTeMoO_6_ photocatalysts.

**Figure 4:**
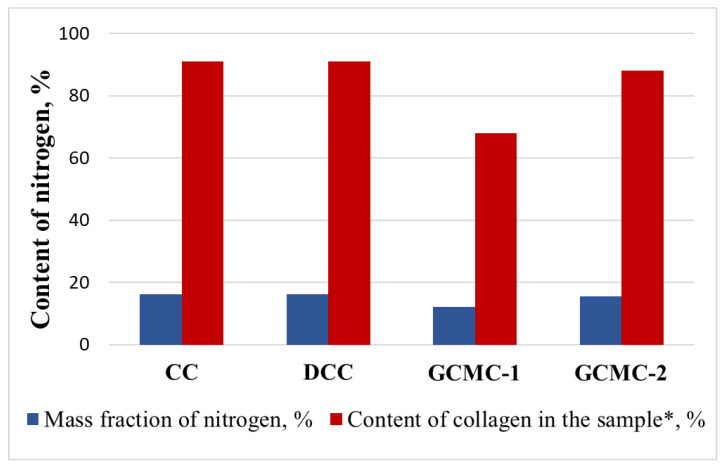
Comparative data on the grafting of MMA onto collagen based on changes in the content of nitrogen using the RbTe_1.5_W_0.5_O_6_ (GCMC–1) and CsTeMoO_6_ (GCMC–2) complex oxides as photocatalysts in comparison with the initial (CC) and dried (DCC) cod collagen. * In terms of collagen according to the well-known formula by multiplying the amount of nitrogen in the sample by the coefficient (5.62). The mass fraction of nitrogen in collagen is a * 5.62 (%), where a is the mass fraction of nitrogen in the sample [55].

In addition, the nitrogen content in GCMC after synthesis significantly decreased compared to the initial collagen sample. The use of the RbTe_1.5_W_0.5_O_6_ and CsTeMoO_6_ compounds in the photocatalytic synthesis led to the content of nitrogen of 12.1 and 15.6%, respectively, which corresponds to the content of collagen in the sample of about 70 and 90% (Figure 4). For the polymer isolated after the synthesis with RbNbTeO_6_, the nitrogen content was about 16.7–17.5%, which corresponds to the initial amount of nitrogen in collagen.

**Figure 5:**
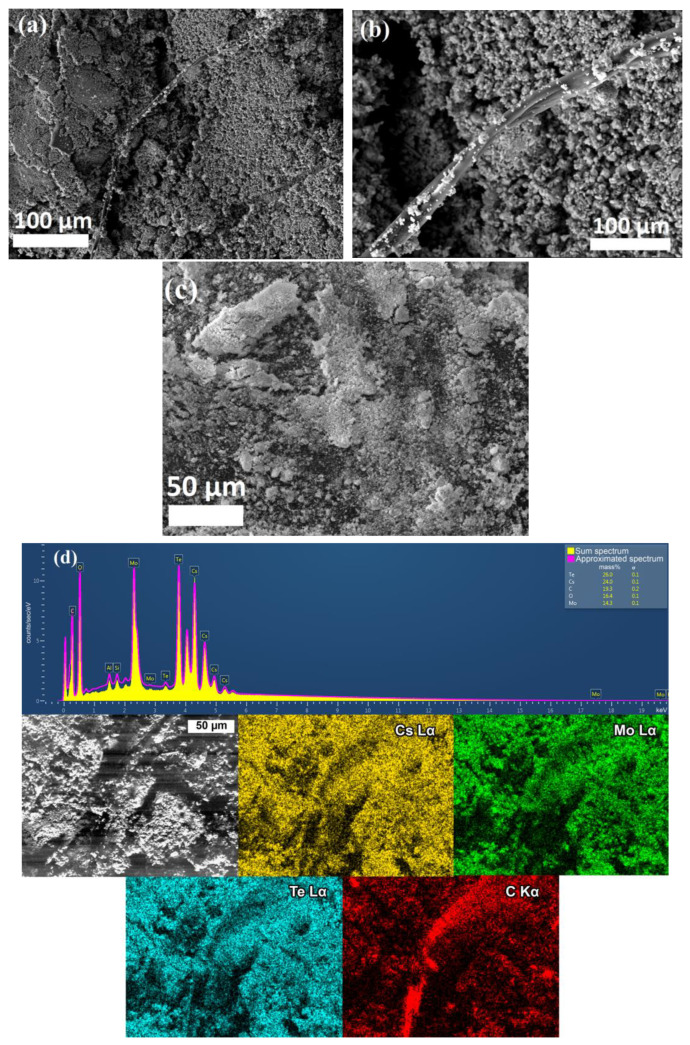
SEM images of the CsTeMoO_6_ photocatalyst after synthesis of the GCMC–2 sample (**a**,**b**), the initial compound (**c**), and EDX-analysis photocatalyst after polymerization (**d**).

In the case of photocatalytic synthesis with RbTeNbO_6_, no fragments of polymer macromolecules were found on the surface of the powder after synthesis, although these polymer fibers were detected for the RbTe_1.5_W_0.5_O_6_ [26] and CsTeMoO_6_ complex oxides (Figure 5a,b,d) by SEM due to the grafting of synthetic polymer fragments onto the surface of β-pyrochlore oxides. The surface of the initial CsTeMoO_6_ photocatalyst was presented for comparison (Figure 5c). The EDX analysis of the CsTeMoO_6_ powder after synthesis confirmed the photocatalyst stability and organic nature of detected fibers, which was presented in elemental maps (Figure 5d).

Thus, the GCMC samples were prepared by photocatalytic synthesis using the RbTe_1.5_W_0.5_O_6_ and CsTeMoO_6_ complex oxides under visible light irradiation (λ = 400–700 nm) at 20–25 °C. The obtained GCMC samples were subjected to enzymatic hydrolysis with pancreatin. At the same time, the RbNbTeO_6_ photocatalyst did not make it possible to obtain noticeable amounts of the grafted synthetic polymer under the reaction conditions.

#### 3.1.3. Features of the Enzymatic Hydrolysis of Graft Copolymer of Methyl Methacrylate with Cod Collagen by Pancreatin

Hydrolysis of GCMC was performed with pancreatin in a neutral medium under conditions described previously [36]. Changes in the molecular weight characteristics of the protein were monitored for 3 days.

**Figure 6:**
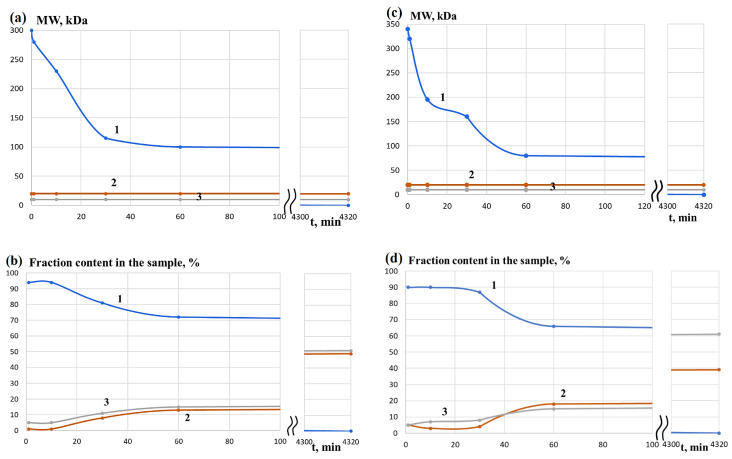
Change in the molecular weight of (**a**) GCMC–1 and (**c**) GCMC–2, as well as proportions of different fractions in hydrolysis of (**b**) GCMC–1 and (**d**) GCMC-2 with pancreatin: 1—fraction with MW above 20 kDa; 2—fraction with MW of about 20 kDa; 3—fraction with MW of about 10 kDa.

As in the case of DCC [49], macromolecules with MW of about 300 kDa rather quickly undergo hydrolysis to peptides with MW of about 20 kDa and predominantly with MW of about 10 kDa (Figure 6). In contrast to DCC, which decomposes predominantly to peptides with MW of about 10 kDa, the ratio of fractions with MW of about 10 kDa and 20 kDa differs much less and their changes are symbatic in the case of GCMC. Moreover, if the content of polymers with MW of more than 20 kDa (~40 kDa) in the products of the enzymatic hydrolysis of DCC is only a few percent after 1 h and completely disappears for 3 days, the content of polymers with MW of more than 20 kDa (~100 and ~80 kDa for GCMC–1 and GCMC–2, respectively) is about 70% after 1 h. Monitoring the process of enzymatic hydrolysis for 3 days indicates a gradual decrease in the content of polymers with MW of more than 20 kDa to almost zero values and a decrease of MW to 20 and 10 kDa.

Within 2–72 h of hydrolysis, there is a uniform decrease in the corresponding parameters. As a result, these data are not shown in figures.

**Figure 7:**
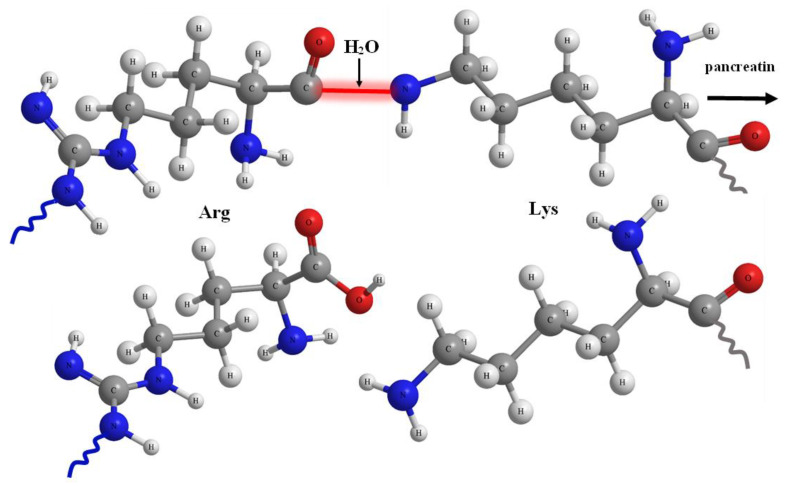
Schematic illustration of proteolytic hydrolysis of the peptide bonds formed by arginine and lysine.

It is well-known that proteolytic enzymes may hydrolyze the protein bonds formed by arginine and lysine (Figure 7) [56]. Apparently, the synthetic fragments grafted to the collagen macromolecule do not prevent the hydrolysis of peptide macromolecules at the same bonds.

The obtained results of the enzymatic hydrolysis of GCMC are important for modeling the process of creating network matrix scaffolds based on them by cross-linking peptides, which are products of enzymatic hydrolysis. It is important that the destruction of GCMC is longer compared to that of collagen. The decrease in the rate of hydrolysis is obviously associated with steric hindrances during the interaction between the active site of the enzyme and the grafted copolymer [57]. The graft copolymers are supposed to be used as precursors for synthesis scaffolds by introducing additional reagents in order to form a stable three-dimensional structure. This allows for the assumption that in the case of scaffolds based on grafted copolymers of cod collagen and synthetic polymers, the biodegradation period will be even longer due to significantly greater steric hindrances. Thus, there will be advantageous differences between grafted synthetic copolymers onto collagen and cod collagen.

## 4. Conclusions

A series of photocatalytic graft copolymerization experiments was carried out using the RbTe_1.5_W_0.5_O_6_, CsTeMoO_6_ and RbNbTeO_6_ complex oxides with β-pyrochlore structure to evaluate their photocatalytic activity. It was demonstrated that the RbTe_1.5_W_0.5_O_6_ and CsTeMoO_6_ materials are capable of photocatalyzing the formation of graft polymers PMMA–collagen from the collagen and MMA mixture under visible light irradiation (λ = 400–700 nm) at 20–25 °C. This was confirmed by GPC and elemental analysis. The use of the RbNbTeO_6_ complex oxide did not allow for the obtainment of noticeable amounts of grafted MMA onto collagen under the reaction conditions.

Moreover, the enzymatic hydrolysis of the resulting graft copolymers was carried out. It was shown that PMMA-collagen graft copolymers rapidly undergo hydrolysis to peptides with MW of about 20 kDa and predominantly with MW of about 10 kDa. In contrast to collagen, which decomposes predominantly to peptides with MW of about 10 kDa, the ratio of fractions with MW of about 10 kDa and 20 kDa differs much less, their changes are symbatic, and the content of polymers with MW of more than 20 kDa is about 70% after 1 h in the case of graft copolymers. Monitoring the process of enzymatic hydrolysis for 3 days indicates a gradual decrease in the content of polymer with MW of more than 20 kDa to almost zero values and a decrease of MW to 20 and 10 kDa. The obtained results allows for the conclusion that synthetic fragments grafted onto the collagen macromolecules do not prevent the hydrolysis of peptide macromolecules but change the rate of polymer degradation. This investigation shows the attractiveness of the prepared materials for the creation of scaffolds in tissue engineering.

The demonstrated advantages of graft copolymers of methyl methacrylate onto collagen, obtained using novel photocatalysts, indicate the possibility of constructing biogels based on them by introducing additional components to obtain three-dimensional structures and test cytotoxicity. These studies could be a continuation of the presented work.

## Data Availability

Data contained within the Article.
